# The Effect of Early vs. Deferred Antiretroviral Therapy Initiation in HIV-Infected Patients With Cryptococcal Meningitis: A Multicenter Prospective Randomized Controlled Analysis in China

**DOI:** 10.3389/fmed.2021.779181

**Published:** 2021-11-19

**Authors:** Ting Zhao, Xiao-lei Xu, Yan-qiu Lu, Min Liu, Jing Yuan, Jing-Min Nie, Jian-Hua Yu, Shui-qing Liu, Tong-Tong Yang, Guo-Qiang Zhou, Jun Liu, Ying-Mei Qin, Hui Chen, Vijay Harypursat, Yao-Kai Chen

**Affiliations:** ^1^Division of Infectious Diseases, Chongqing Public Health Medical Center, Chongqing, China; ^2^Division of Infectious Diseases, Xixi Hospital of Hangzhou, Zhejiang, China; ^3^Division of Infectious Diseases, Guiyang Public Health Clinical Center, Guizhou, China; ^4^Division of Infectious Diseases, Public Health Clinical Center of Chengdu, Sichuan, China; ^5^Division of Infectious Diseases, The First Hospital of Changsha, Hunan, China; ^6^Division of Infectious Diseases, Kunming Third People's Hospital, Yunnan, China; ^7^Division of Infectious Diseases, The Fourth's Hospital of Nanning, Guangxi, China; ^8^School of Biomedical Engineering, Capital Medical University, Beijing, China

**Keywords:** HIV, IRIS, antiretroviral therapy, mortality, cryptococcal meningitis

## Abstract

**Background:** The optimal timing for initiation of antiretroviral therapy (ART) in HIV-positive patients with cryptococcal meningitis (CM) has not, as yet, been compellingly elucidated, as research data concerning mortality risk and the occurrence of immune reconstitution inflammatory syndrome (IRIS) in this population remains inconsistent and controversial.

**Method:** The present multicenter randomized clinical trial was conducted in China in patients who presented with confirmed HIV/CM, and who were ART-naïve. Subjects were randomized and stratified into either an early-ART group (ART initiated 2–5 weeks after initiation of antifungal therapy), or a deferred-ART group (ART initiated 5 weeks after initiation of antifungal therapy). Intention-to-treat, and per-protocol analyses of data for these groups were conducted for this study.

**Result:** The probability of survival was found to not be statistically different between patients who started ART between 2–5 weeks of CM therapy initiation (14/47, 29.8%) vs. those initiating ART until 5 weeks after CM therapy initiation (10/55, 18.2%) (*p* = 0.144). However, initiating ART within 4 weeks after the diagnosis and antifungal treatment of CM resulted in a higher mortality compared with deferring ART initiation until 6 weeks (*p* = 0.042). The incidence of IRIS did not differ significantly between the early-ART group and the deferred-ART group (6.4 and 7.3%, respectively; *p* = 0.872). The percentage of patients with severe (grade 3 or 4) adverse events was high in both treatment arms (55.3% in the early-ART group and 41.8% in the deferred-ART group; *p*=0.183), and there were significantly more grade 4 adverse events in the early-ART group (20 vs. 13; *p* = 0.042).

**Conclusion:** Although ART initiation from 2 to 5 weeks after initiation of antifungal therapy was not significantly associated with high cumulative mortality or IRIS event rates in HIV/CM patients compared with ART initiation 5 weeks after initiation of antifungal therapy, we found that initiating ART within 4 weeks after CM antifungal treatment resulted in a higher mortality compared with deferring ART initiation until 6 weeks. In addition, we observed that there were significantly more grade 4 adverse events in the early-ART group. Our results support the deferred initiation of ART in HIV-associated CM.

**Clinical Trials Registration:** www.ClinicalTrials.gov, identifier: ChiCTR1900021195.

## Introduction

Early initiation of antiretroviral therapy (ART) has been found to decrease mortality from a number of opportunistic infections (OIs) associated with HIV infection, such as Pneumocystis pneumonia and tuberculosis (TB) (1). However, whether early ART initiation provides a similar clinical benefit for HIV-positive patients with cryptococcal meningitis (CM) remains unclear. For HIV patients with CM, the challenges of early ART initiation include adverse reactions, overlapping toxicities of ART and antifungal therapy, and the development of immune reconstitution inflammatory syndrome (IRIS) (2, 3). Deferred initiation of ART may increase the risk of disease progression, which could lead to death. Clinicians must therefore tread a very fine line when choosing the precise timing of ART initiation for these patients.

The Infectious Diseases Society of America's 2010 guidelines recommend that ART in patients with CM should be started between 2 and 10 weeks after antifungal therapy has been initiated (4, 5). However, it is not clear precisely when to start ART during the prescribed period of between 2 and 10 weeks. In affluent countries, some experts may delay ART by up to 10 weeks (i.e., after completion of consolidation fluconazole therapy) in patients who have close follow-up and access to treatment that may prevent further OIs. A prospective randomized controlled trial in Zimbabwe observed that patients who received ART within 72 h of diagnosis of CM had a mortality rate of 88%, compared to 54% in those who delayed ART initiation to 10 weeks after initiation of antifungals, with all participants receiving 800 mg of fluconazole daily for CM induction therapy (6). The deferred-ART strategy minimizes the risk of development of paradoxical IRIS and drug-drug interactions between ART drugs and high-dose antifungal therapy. However, this approach is not universally applicable since the benefits of a more rapid immune recovery in resource-limited areas usually outweigh the risk of development of IRIS (7).

The classic Cryptococcal Optimal ART Timing (COAT) prospective study showed that deferring ART initiation until 5 weeks after the diagnosis of CM, was associated with significantly improved survival as compared with initiating ART at 1–2 weeks (8). One early study found that compared with deferred ART (median time to ART initiation was 32 days), early ART (median time to ART initiation was 7 days) was not associated with improved cerebrospinal fluid (CSF) fungal clearance, and resulted in a higher risk of development of IRIS (3). Additionally, observations from meta-analytic data sets have also indicated a higher probability of survival among people who initiate ART until 4 weeks after CM diagnosis (9, 10). Thus, for individuals in resource-limited settings, ART should be initiated between 4 and 6 weeks after the initial diagnosis of CM, because the benefits of a faster immune recovery usually outweigh the risks associated with development of IRIS. This approach is consistent with guidelines from the World Health Organization (7). However, the 2018 guidelines from the International Antiviral Society-USA panel suggest an earlier initiation of ART in these patients under certain circumstances (11).

The optimal timing of ART initiation thus remains somewhat controversial for HIV patients with CM. We have conducted this multicenter prospective study in order to determine the optimal timing for ART initiation in ART-naive HIV-infected patients newly diagnosed with CM in China.

## Methods

### Study Design

The present study was an open-label, multicenter, prospective, randomized, and controlled clinical trial with two treatment arms: an early-ART arm (with ART initiated within 2–5 weeks of commencing antifungal therapy) and a deferred ART arm (with ART initiated after 5 weeks of antifungal therapy). This study was approved by the Ethics Committee of Chongqing Public Health Medical Center (Approval Number: 2019-003-02-KY) and was duly registered as one of 12 trials under a general project, at the Chinese Clinical Trial Registry (ChiCTR1900021195).

Patients were enrolled at the following seven medical centers in China: Chongqing Public Health Medical Center, Public Health Clinical Center of Chengdu, Guiyang Public Health Clinical Center, Kunming Third People's Hospital, the First Hospital of Changsha, Xixi Hospital of Hangzhou, and the Fourth People's Hospital of Nanning.

### Study Participants

Recruitment to the trial began in January 2019 and ended in December 2020. Each participant was followed for up to 24 weeks. Eligible participants had HIV infection (≥18 years old), a clinical syndrome consistent with a diagnosis of CM, and microbiological confirmation of disease, as indicated by one or more of the following test results: positive India ink staining of CSF, culture of cryptococcus species from CSF or blood, or cryptococcal antigen detection in CSF. Participants were excluded if they had previously been exposed to ART, were allergic or intolerant to any of the therapeutic drugs, had severe concurrent CNS infections, were pregnant or lactating, had serious coexisting conditions, were intravenous drug users, or were not willing to provide written informed consent.

An alanine aminotransferase (ALT) concentration that was more than five times the upper limit of the normal range, a neutrophil count less than 500 per cubic millimeter, a polymorphonuclear leukocyte count less than 1,000 per cubic millimeter, hemoglobin levels below 60 grams per liter, a platelet count less than 50,000 per cubic millimeter, or an elevated creatinine level more than 1.5 times the upper limit of the normal range were exclusion criteria (i.e., a patient who met one or more of these criteria at baseline was withdrawn from the trial).

### Treatment Regimens

All of the enrolled patients received conventional drug treatments for both CM and HIV in accordance with the recommendations of the Chinese Guidelines for the Diagnosis and Treatment of HIV/AIDS (2018) (12).

#### Antifungal Therapy

In light of local Chinese guidelines concerning management of HIV/CM, we used amphotericin B deoxycholate combined with flucytosine during the induction phase of treatment. Fluconazole or voriconazole could also be used as alternative therapeutic drugs at the induction phase. The attending physician had the discretion to adjust drug regimens based on the patient's clinical condition.

#### ART Therapy

In accordance with local guidelines, the preferred antiretroviral regimen of tenofovir disoproxil fumarate (TDF) (300 mg/d) + lamivudine (3TC) (300 mg/d) + efavirenz (EFV) (600 mg/d) was used for the treatment of HIV infection, while other ART regimens were also optional alternatives.

### Randomization and Follow-Up

Using a computer-generated sequence of random numbers, participants were randomly assigned to the early-ART or the deferred-ART arms of the study, at an allocation ratio of 1:1. After randomization, clinicians taking care of participants were not blinded to treatment allocation. Study visits, at which clinical symptoms and routine laboratory tests were assessed, took place at baseline, and at 4, 8, 12, and 24 weeks after randomization.

### Study Outcomes

The primary outcome was the mortality rate and incidence of paradoxical CM-related IRIS (CM-IRIS) at week 24. Secondary outcome measures included 5 week, 5–10 week, and 10–24 week all-cause mortality, fungal clearance, CD4+ T-cell counts, virologic suppression (<50 copies/mL) at 24 weeks, and adverse events (grade 3–4). CM-IRIS was defined in accordance with the published case definition (13). Adverse events were graded from grade 3 (moderate) to grade 4 (severe) according to the Division of AIDS Table for Grading the Severity of Adult and Pediatric Adverse Events: December 2017.

### Statistical Analyses

Primary and secondary outcome analyses were performed in both the intention-to-treat analysis set, and the per-protocol analysis set, respectively. The per-protocol population excluded major protocol violators, patients who withdrew from the study, and those who were lost to follow-up. The time to primary outcome was estimated by the Kaplan-Meier method, and any differences between two groups were evaluated with a stratified log-rank test. Cox proportional-hazards regression analysis was used to estimate the hazard ratio, with 95% confidence intervals. We used the same methods in the subgroup analysis defined according to baseline characteristics. For comparison of other proportions between the two groups, we used Pearson χ^2^ test, or Fisher's exact test. Continuous variables were compared using the Mann–Whitney *U* test or Student's t-test. All analyses were performed using Stata software (StataCorp, College Station, Texas, USA), Version 16, and Statistical Package for the Social Sciences (SPSS) software, Version 24 (IBM-SPSS, Armonk, New York, USA).

## Results

### Participants

**Figure 1:**
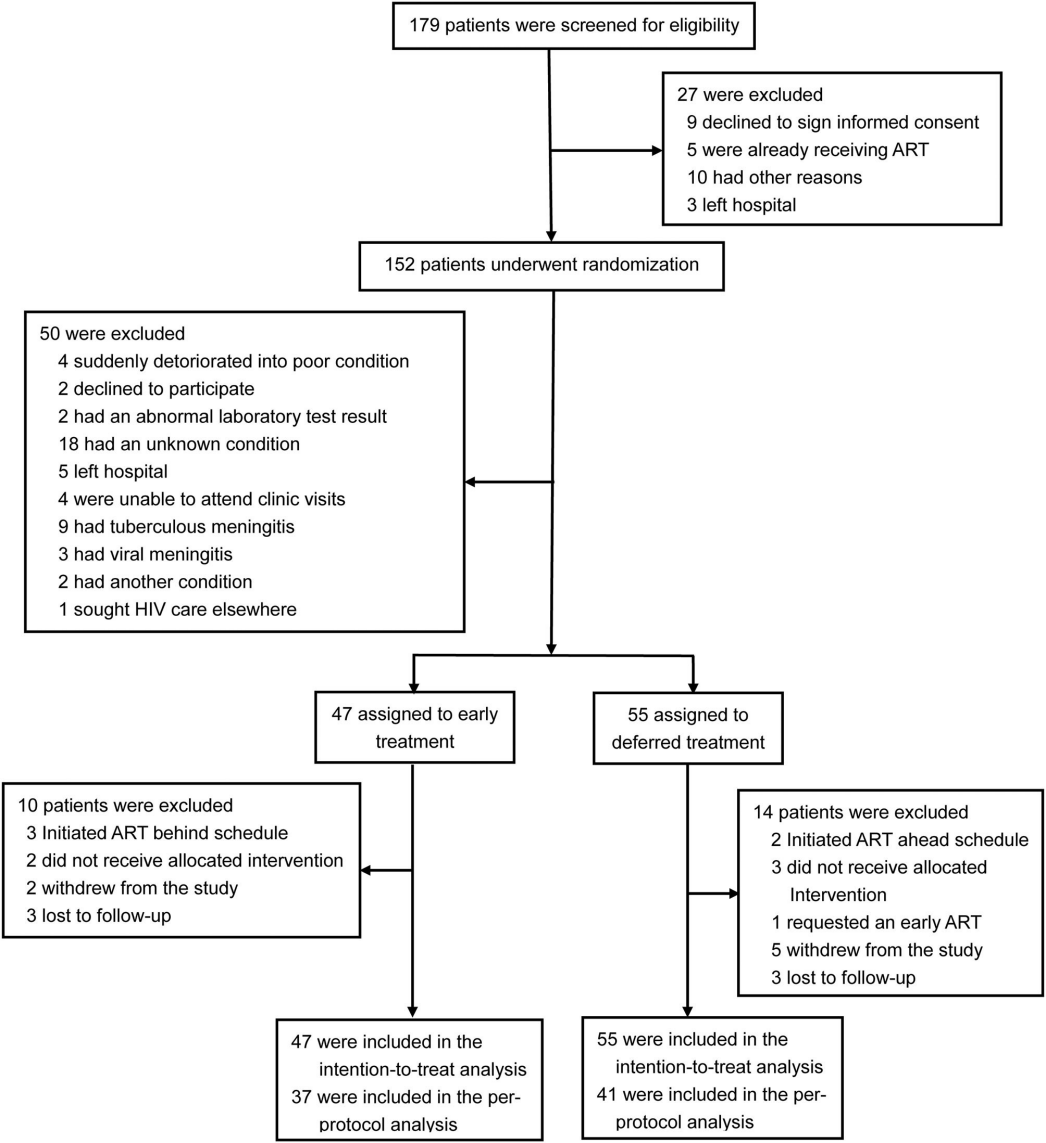
The flow diagram of the study.

A total of 179 patients were screened for eligibility, 102 participants with CM were enrolled in the trial and randomly assigned to early ART (47 patients) or deferred ART (55 patients), and 24 patients (ten patients in the early-ART group and fourteen patients in the delayed-ART group) were excluded from the per-protocol analysis upon further analysis. A flow diagram detailing the differences between the intention-to-treat population as compared to the per-protocol population is shown in Figure 1.

The median time from diagnosis of CM to ART initiation was 27 days (IQR, 22–32) in the early-ART group and 38 days (IQR, 36–47) in the deferred-ART group. In the early-ART group, two participants died after randomization but before the initiation of ART. In the deferred-ART group, three participants died after randomization and before ART-initiation, and thus never received ART. All five participants were included in the intention-to-treat analysis. There were no significant differences between the two arms with respect to initial ART regimens used. Integrase inhibitors (INSTIs) were used in 62.2% (28/45) of subjects in the early-ART arm and 67.3% (35/52) of subjects in the deferred-ART arm, while non-nucleoside reverse transcriptase inhibitor (NNRTI)-based regimens with two nucleoside reverse transcriptase inhibitors (NRTIs) were used in 26.7 and 25% of subjects in each arm, respectively. Only 5 subjects received a boosted protease inhibitor (PIs) with two NRTIs (three subjects in the early-ART arm and two subjects in the delayed-ART arm); the remaining four subjects (two allocated to the early-ART treatment group and two assigned to the deferred-ART treatment group) initiated fusion inhibitor (FI)-based regimens.

### Baseline Characteristics

The demographic and baseline clinical characteristics for the 102 study subjects were well balanced across the two study arms (Table 1, Supplementary Table 1). Subjects were primarily men (75.5%), with an average age of 45.5 years. The baseline median CD4+ T-cell count was 28 cells/μL (interquartile range, IQR, 12–54), and 70% of the subjects entered the study with a CD4+ T-cell count of < 50 cells/μL. The median plasma viral load was 5.6 log_10_ copies/mL (IQR, 5.0–5.9).

**Table 1:** Baseline characteristics according to treatment group.

**Characteristic**	**Deferred ART (*n* = 55)**	**Early ART (*n* = 47)**	***p*-value**
Age, median years (IQR)	44.5 ± 13.3	46.7 ± 12.9	0.388
Male, sex, n (%)	42 (76.4%)	35 (74.5%)	0.824
BMI, mean ± SD	20.1 (17.7, 21.5)	19.7 (18.1, 21.9)	0.588^*^
**Symptom**			
Headache, *n* (%)	42 (76.4%)	32 (68.1%)	0.350
Fever, *n* (%)	35 (63.6%)	28 (59.6%)	0.674
Nausea, *n* (%)	26 (47.3%)	23 (48.9%)	0.867
Vomiting, *n* (%)	22 (40%)	22 (46.8%)	0.489
Impaired consciousness, *n* (%)	13 (23.6%)	10 (21.3%)	0.776
Stiff neck, *n* (%)	24 (43.6%)	18 (38.3%)	0.585
**Routine blood tests**			
WBC, median × 10^9^/L(IQR)	4.4 (3.1, 5.5)	3.9 (2.8, 5.5)	0.466^*^
Hemoglobin, mean ×g/L ± SD	109.8 ± 22.4	105.2 ± 20.3	0.288
Platelets, mean × 10^9^/L) ± SD	171.1 ± 68.9	165.0 ± 76.5	0.674
**Blood biochemistry**			
TBIL, median μmol/L(IQR)	7.9 (6.4, 11.3)	9.3 (7.5, 14.2)	0.735^*^
ALT, median U/L(IQR)	24.0 (14.4, 34.0)	26.0 (17.0, 45.0)	0.293^*^
AST, median U/L(IQR)	27.0 (17.0, 37.0)	24.0 (18.0, 37.0)	0.677^*^
Urea, median mmol/L(IQR)	4.5 (3.2, 5.6)	4.0 (3.1, 5.7)	0.769^*^
Creatinine, median μmol/L(IQR)	56.0 (49.0, 70.1)	57.5 (51.0, 69.0)	0.822^*^
Other OIs			
Pulmonary tuberculosis, *n* (%)	8 (14.5%)	5 (10.6%)	0.555
Pneumocystis pneumonia, *n* (%)	5 (9.1%)	4 (8.5%)	1.0^#^
Cytomegalovirus infection, *n* (%)	7 (12.7%)	4 (8.5%)	0.494
**CSF profile**			
ICP, median mm H_2_O (IQR)	314 (198.8, 385)	300 (230, 370)	0.981^*^
CSF WBC, median × 10^6^/L (IQR)	23.5 (6.0, 73.5)	10.5 (4.0, 66.3)	0.434^*^
CSF glucose level, median mmol/L (IQR)	2.5 (1.5, 3.4)	2.4 (1.4, 2.8)	0.414^*^
CD4, median cells/μL (IQR)	34.5 (11.8, 59.5)	25.0 (12.0, 44.0)	0.327^*^
CD4/CD8	0.08 (0.05, 0.15)	0.09 (0.04, 0.18)	0.881^*^
HIV RNA, log10 copies/mL (IQR)	5.5 (5.0, 5.9)	5.6 (5.1, 5.9)	0.516^*^
**Treatment strategy**, ***n*** **(%)**			
ART regimens containing INSTIs	33 (60%)	28 (59.6%)	0.965
AmB/LipAmB as induction antifungal therapy	42 (76.4%)	40 (85.1%)	0.268

*Data are present as n (%), mean (± SD) for normally distributed data, or median (IQR) for non-normally distributed data. p-values from χ^2^ tests for categorical variables, and Student's t-tests for continuous variables, unless otherwise specified*.

* *Mann-Whitney U tests was used*.

# *indicates calculations using the Fisher exact test*.

### Primary Outcomes

**Figure 2:**
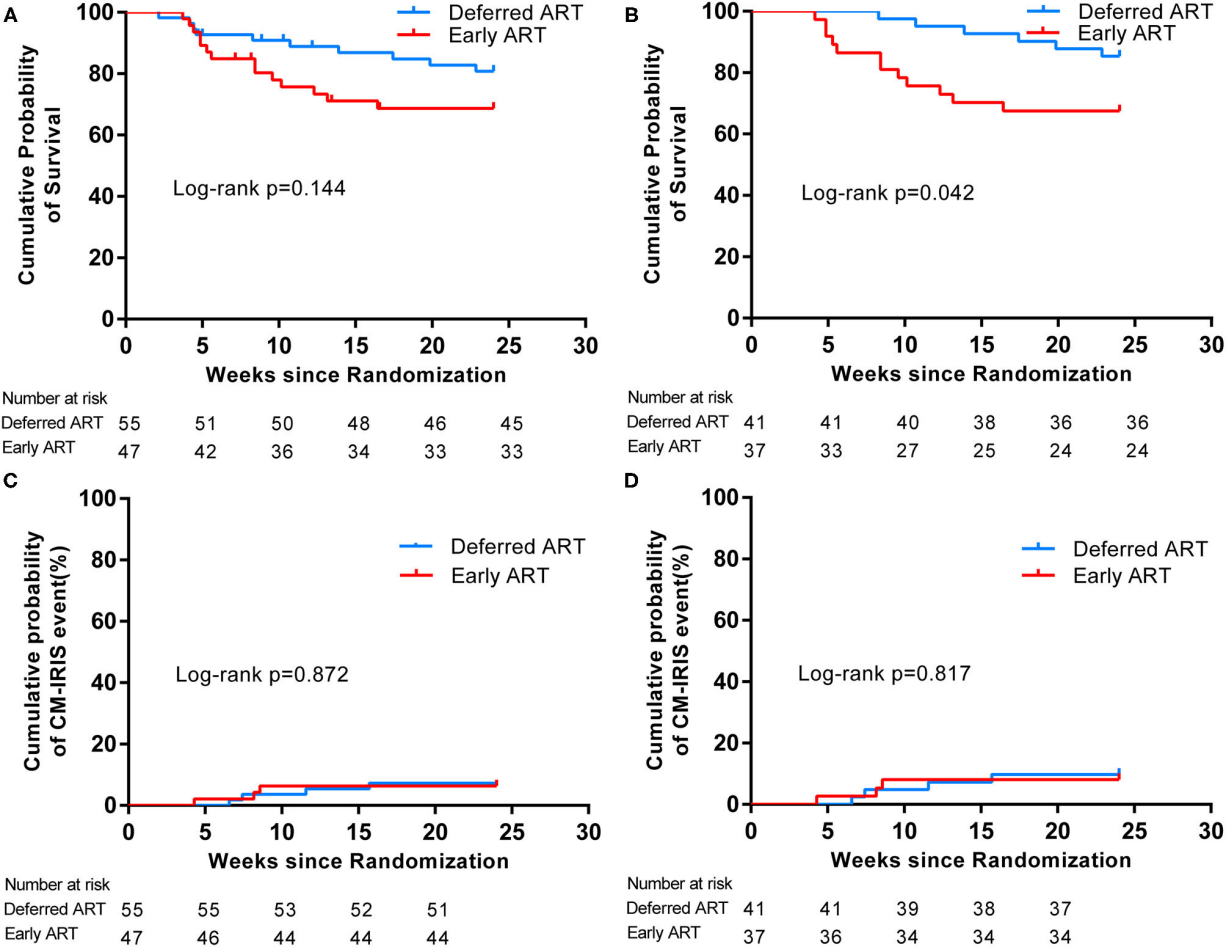
Kaplan-Meier survival estimates of primary outcomes according to timing of ART. Cumulative probability of survival from randomization (time = 0) to 24 weeks in the Intention-to-Treat Population **(A)** and in the Per-Protocol Population **(B)**. Cumulative rates of CM-IRIS events in the Intention-to-Treat Population **(C)** and in the Per-Protocol Population **(D)**.

In the intention-to-treat population, 10 (18.2%) subjects assigned to the deferred treatment group succumbed, compared with 14 (29.8%) subjects in the early antiretroviral treatment group. Upon statistical analysis, no significant difference in the 24-week time distribution of survival were found among the two treatment groups (*p* = 0.144 by log-rank test). The hazard ratio for mortality in the deferred-ART group was 0.820 (95% confidence interval [CI], 0.625, 1.075), compared with the early-ART group (Figure 2A). Importantly, a difference in mortality between the two study arms was observed at 5–10 weeks after enrolment (*p* = 0.043). Mortality did not differ significantly before or after this period (Table 2).

**Table 2:** Secondary outcomes in the two treatment arms in the intention-to-treat population and in the per-protocol population.

	**Intention-to-treat population**			**Per-protocol population**		
**Event-no. of patients (%)**	**Deferred ART**	**Early ART**	***p*-value**	**Deferred ART**	**Early ART**	***p*-value**
	**(*n* = 55)**	**(*n* = 47)**		**(*n* = 41)**	**(*n* = 37)**	
**Mortality**						
0–5W	4/55	5/47	0.729^#^	0/41	3/37	0.046^#^
5W−10W	1/51	6/42	0.043^#^	1/41	6/34	0.042^#^
10W−24W	5/50	3/36	1.0^#^	5/40	3/27	1.0^#^
CSF culture positive at 14 days of CM therapy	30/52	22/44	0.424	19/39	17/35	0.990
CSF culture positive at 10 weeks of CM therapy	13/52	8/44	0.421	5/39	6/35	0.602
Positive India ink staining of CSF at 10 weeks of CM therapy	21/40	7/25	0.052	11/28	5/18	0.424
CD4+ count at 24 weeks (median change from baseline) (IQR)	80 (48, 129)	61 (32,114)	0.729	80 (48, 129)	66 (32, 123)	0.785^*^
Plasma HIV RNA < 50 copies/mL at 24 weeks	12/20	7/15	0.433	11/19	7/15	0.515
Laboratory Adverse Events Grades 3–4	19/55	21/47	0.296	16/41	19/37	0.274
Clinical Adverse Events Grades 3–4	6/55	10/47	0.151	5/41	10/37	0.176
Grade 3 adverse events	16/55	17/47	0.446	16/41	16/37	0.705
Grade 4 adverse events	13/55	20/47	0.042	10/41	19/37	0.014
Total events	23/55	26/47	0.174	23/41	26/37	0.196

*Data are n (%), or median (IQR)*.

# *indicates calculations using Fisher's exact test; otherwise, figures were calculated using Pearson's χ^2^ test*.

* *Mann-Whitney U test was used*.

CM-IRIS events occurred in three subjects in the early-ART group and in four subjects in the deferred-ART group, and the rates of IRIS events in the two groups did not differ significantly during the follow-up period (*p* = 0.872). The estimated hazard ratio for IRIS events (early vs. deferred treatment) was 1.042 (95% CI 0.632–1.716), as shown in Figure 2C.

In the per-protocol population, the proportion of subjects who succumbed by 24 weeks was greater in the early-ART group than in the deferred-ART group [12 of 37 (32.4%) vs. 6 of 41 (14.6%)] (Figure 2B). The difference in overall survival between the two groups was calculated to be statistically significant (*p* = 0.042 by log-rank test). The risk of death in the deferred-ART group was 0.722 times as low as the early-ART group (95% CI, 0.521–1.001). The difference in mortality between the two groups occurred at 5–10 weeks after enrolment. There were seven deaths during this period; one in the deferred-treatment group and six in the early-treatment group (*p* = 0.042).

The incidence of CM-IRIS over 24 weeks was 8.1% in the early-treatment group, compared with 9.8% in the deferred-treatment group (Figure 2D). Again, no significant difference in the incidence of IRIS was observed between the two study groups. The hazard ratio of IRIS events in the early-ART arm was 1.061 (95% CI 0.644–1.747) compared with the delayed-ART arm.

### Subgroup Analyses

**Figure 3:**
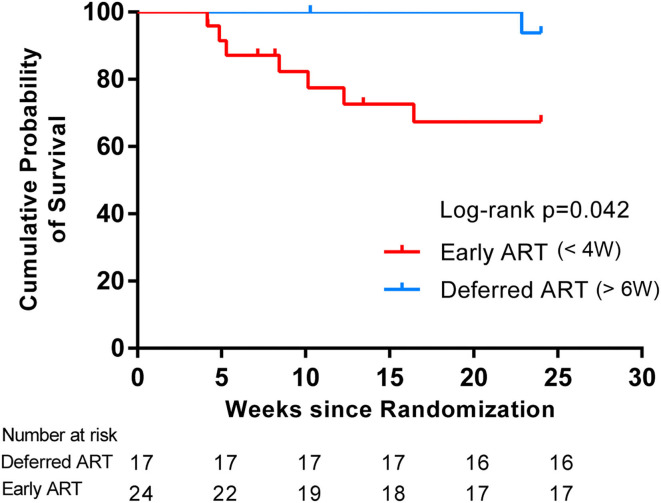
Kaplan-Meier curves for survival rates and timing of ART initiation. The subgroup analysis was assessed to determine whether stratification of ART initiation timing had an effect on survival in each treatment group. Initiating ART within 4 weeks after the diagnosis of CM resulted in a higher mortality compared with deferring ART initiation until 6 weeks, suggesting a differential response to the timing of ART initiation.

We assessed the interactions between the six prognostic factors (sex, age, intracranial pressure, CSF white cell count, HIV RNA, and CD4+ T-cell count) and the treatment prescribed. As shown in Table 3, there were no significant associations between any of the individual factors and the treatment. In addition, comparisons of the exact antifungal treatment regimens that patients received and the actual time of ART initiation between the two study groups provided no evidence to guide exactly when to initiate ART (Table 3). The difference in survival rate between patients who started ART within 4 weeks of antifungal treatment in the early-ART group and those who delayed ART initiation until 6 weeks after antifungal treatment in the deferred-ART group was then further analyzed. The results indicated that initiating ART within 4 weeks after the diagnosis of CM was significantly associated with higher mortality compared with deferring ART initiation until 6 weeks (Figure 3).

**Table 3:** Subgroup analysis of 24-week mortality in the Intention-to-Treat Population.

	**Deferred ART**		**Early ART**		**Hazard ratio (95% CI)**	***p*-value**
	No. of patients	No. of deaths	No. of patients	No. of deaths		
**Age (years)**						
<50	33	5	27	6	0.873 (0.588, 1.297)	0.502
≥50	22	5	20	8	0.778 (0.536, 1.131)	0.189
**Sex**						
Male	42	8	35	10	0.853 (0.625, 1.162)	0.313
Female	13	2	11	4	0.757 (0.429, 1.334)	0.335
**ICP at meningitis (mmH**_**2**_ **O)**						
<250	18	2	12	2	0.871 (0.452, 1.676)	0.679
≥250	37	8	34	12	0.838 (0.622, 1.129)	0.246
**CSF white cell count at meningitis (10** ^**6**^ **/L)**						
<20	25	6	26	6	0.997 (0.684, 1.454)	0.998
≥20	30	4	20	8	0.673 (0.451, 1.004)	0.053
**CD4 cell count at randomization(cells/μL)**						
<50	36	9	36	12	0.890 (0.667, 1.188)	0.429
≥50	19	1	11	2	0.641 (0.288, 1.428)	0.227
**HIV RNA (log10 copies/mL)**						
<5.5	28	6	18	5	0.871 (0.586, 1.295)	0.494
>5.5	27	4	29	9	0.765 (0.516, 1.133)	0.181
**Induction treatment**						
AmB+5FC	21	4	24	6	0.910 (0.597, 1.387)	0.661
FCZ±5FC	4	0	3	0	-	-
AmB+5FC+FCZ	5	2	4	2	0.918 (0.799, 1.770)	0.799
AmB+ FCZ	12	1	10	3	0.580 (0.272, 1.234)	0.157
VCZ+ AmB	4	0	2	1	-	-
VCZ+5FC	4	1	3	1	1.145 (0.430, 3.050)	0.787
Other	5	2	1	1	0.607 (0.241, 1.531)	0.290
**Timing for initiating ART**						
2W−3W	0	0	9	3	-	-
3W−4W	1	0	15	4	-	1
4W−5W	2	1	18	5	1.663 (0.778, 3.556)	0.189
5W−6W	32	5	1	0	-	-
> 6W	17	1	2	0	-	-
NA	3	3	2	2	0.699 (0.312, 1.567)	0.385

*NA indicates that ART was not initiated prior to death*.

### Secondary Outcomes

In the intention-to-treat population, the proportion of patients with CSF culture positivity at 14 days in the early-ART group and in the deferred-ART group were 50% (22/44), and 57.5% (30/52), respectively, and the difference was calculated to be statistically insignificant between the two groups. The median change in CD4+ T-cell counts from baseline to 24 weeks was 61 (32,114) vs. 80 (48,129) in the early vs. deferred treatment arms, respectively (*p* = 0.729 for both comparisons). Both treatment arms eventually reached similar CD4+ T-cell counts by week 24, and the median CD4+ T-cell count at week 24 was 122.5 cells/μL in the early-ART arm, compared to 116 cells/μL in the deferred-ART arm (data not shown). Virological control differed between the two arms; 7 early-ART subjects and 12 deferred-ART subjects achieved a viral load of 50 copies/mL at week 24 based on a standard intention-to-treat analysis (Table 2).

During follow-up, 82 grade 3 or 4 adverse events were recorded in 49 patients. Adverse events (grade 3 or 4) were reported among 26 patients assigned to the deferred-ART strategy, and among 23 patients assigned to the early-ART strategy. No significant differences were observed in the number of patients with laboratory or clinical adverse events between the study groups. However, significantly more patients in the early ART group experienced grade 4 adverse events (20 vs. 13; *p* = 0.042). Five (10.6%) and three (5.5%) patients in the early and deferred ART groups, respectively, experienced one or more neurological events (grade 3 or 4); there was no significant association with the treatment arm (*p* = 0.465) (Supplementary Table 2). The frequency of grade 3 or 4 anemia events appeared to be higher in the early-ART group than in the deferred ART group, but this difference was calculated to not be statistically significant (25.5 vs. 10.9%; *p* = 0.053) (Supplementary Table 2).

In the per-protocol population, the timing of ART initiation had no effect on virtually all secondary end points (Table 2). These results were similar to those of the intention-to-treat analysis. The rate of CSF culture positivity at 14 days of amphotericin B therapy was almost identical in the two treatment arms. CD4+ T-cell counts and virological responses did not differ significantly at 24 weeks. The frequency and rates of adverse events did not differ significantly between the two treatment groups. A total of 23 subjects (56.1%) in the deferred arm and 26 subjects (70.3%) in the early arm experienced one or more grade 3 or 4 adverse events over the 24 weeks of follow-up. We also found that the proportion of patients with grade 4 adverse events was significantly higher in the early-ART group compared to the deferred-ART group (*p* = 0.014), as shown in Table 2.

## Discussion

Currently, there remains uncertainty regarding the optimum timing of ART initiation in HIV-infected patients presenting with CM. The uncertainty relates to determining precisely when to initiate ART after a diagnosis of CM, and involves balancing the survival benefits conferred by ART against the risk of developing IRIS. Previous studies in mixed patient cohorts or non-randomized studies have suggested that early ART treatment could decrease mortality and possibly improve clinical outcomes (1, 3). However, a systematic review which included subjects from the United States, South Africa, Zimbabwe, and Puerto Rico found insufficient evidence to support either early or deferred initiation of ART (14). In another large Latin-American cohort study, there also appeared to be no statistically significant difference in the probability of survival between patients who started ART within 2 weeks of CM vs. those initiating ART between 2 and 8 weeks after CM diagnosis (15).

In contrast to these findings, a recent review of four randomized controlled trials suggested a higher risk of mortality among people who initiate ART within four weeks of CM diagnosis (10). Of these four trials, a trial from Zimbabwe showed decreased survival in individuals with HIV/CM who were started on ART within 72 h vs. after 10 weeks from initial CM treatment (6). A classic trial conducted in Uganda and South Africa found that patients randomized to start ART within 2 weeks after diagnosis of CM had a higher risk of mortality compared to those assigned to deferred ART until 5 weeks after diagnosis of CM (8). However, both of the preceding studies were terminated early after observing increased mortality in the early ART arm, and may thus have overestimated the size of the effect of early ART on mortality (16). Another clinical trial, showing the opposite effect, found fewer deaths and AIDS progression events in those treated with early vs. deferred-ART (1). Conversely, a study conducted in Botswana failed to demonstrate a reduction in mortality with early ART.

In the present study, data from the intention-to-treat analysis found that initiating ART between 2 and 5 weeks of CM treatment showed a trend toward decreased survival in patients with HIV-associated CM, as compared with deferring ART until 5 weeks after the start of CM therapy. Moreover, the outcomes comparing early initiation of ART (<4 weeks after starting antifungal treatment) with deferred initiation of ART (6 weeks after starting antifungal treatment) suggested that earlier ART was indeed detrimental to the outcomes of patients with concurrent CM and HIV infection; this association reached statistical significance in the subgroup analysis for the primary outcome. Possible reasons for the detrimental effects of early ART in these participants include progression of cryptococcal disease, inadequate CSF pressure management, combined drug interactions, and additive toxic drug effects (17). Also, since neurological deterioration often occurred unexpectedly, it was rarely possible to perform appropriate investigations in order to establish the cause for the deterioration. We thus postulated that the higher short-term mortality observed with early ART initiation may relate to adverse consequences in the CNS. Potential causes are likely to involve either occult paradoxical immunological mechanisms, overt CNS IRIS, or the unmasking of a different comorbid CNS opportunistic infection, each occurring in the context of early initiation of ART.

More than half of the patients in research settings had succumbed by 10 weeks after starting antifungal therapy, even among patients primarily treated with amphotericin-based therapy. The results emphasize the high acute mortality in patients with CM; these acute deaths were attributed mainly to CM or complications related to CM. The findings observed in the present study are consistent with what has been observed in other studies and cohorts (6, 8). Importantly, in our subgroup analysis, between-group differences were found in the early-ART group compared to the deferred-ART group with respect to the rate of mortality during the 5-10 weeks after enrolment. The results of analyses performed in the per-protocol population were also consistent with this result. The risk factors for mortality, in addition to high fungal burden, were slow clearance of infection, altered mental status, older age, low CD4+ T-cell count, low weight, and anemia, which have all been previously identified as predictors of mortality in HIV cohorts in general (18). Cell-mediated immunity has been observed to play an important role in the control of *Cryptococcus* infection; low CSF white cell counts and low levels of IFN-γ, TNF-α, and IL-6 were shown to be inversely associated with survival (19). The higher short-term mortality trend observed with early ART initiation in our trial may thus be related to a poor cellular inflammatory responses at the site of infection in the CNS.

The early-ART group appeared to be associated with a higher frequency of severe (grade 4) adverse events, suggesting that early ART initiation may actually be disadvantageous in terms of adverse events. This increase in adverse events could not be accounted for by differences in ART regimens used, as the differences in adverse events at baseline were similar between the two groups. Severe anemia appeared to occur more commonly in the early ART group; however, this association was calculated to not be statistically significant. The adverse consequences related to early ART initiation after cryptococcosis diagnosis may involve unrecognized increases in inflammatory responses in the CNS due to ART-associated immune reconstitution in the first month after ART initiation. Interestingly, we observed no significant increase in specific neurological events in the early ART group. These results concur with those of a past randomized controlled study that compared early-vs. deferred-ART in patients with HIV-associated tuberculous meningitis (20). In contrast to these results, however, early ART initiation is considered to be beneficial in other non-central nervous system (non-CNS) opportunistic infections, and has been demonstrated to reduce AIDS-defining events and deaths on these cohorts (1, 21). Thus, the consequences of early ART initiation in patients with CNS infections, such as cryptococcal and tuberculous meningitis, seem to differ from those of early ART initiation in non-CNS opportunistic infections.

Early initiation of ART most likely resulted in the increased rates of CM-IRIS and the excess mortality observed in the early ART group. Interestingly, however, there was no statistically significant increase in paradoxical CM-IRIS incidence in the present study after early ART initiation (6.4%) when compared to deferred ART (7.3%) (*p* = 0.872). Moreover, the overall incidence of IRIS was low, despite most subjects having an advanced immunodeficient immunological state. Our observation that the timing of ART initiation is positively associated with the incidence of CM-IRIS is consistent with some (1, 22), but not other (23–25) studies. One possible reason for this is that the case definition of paradoxical CM-IRIS relies on the requirement for previous clinical improvement of CM (13). In our study, clinicians were often unable to accurately distinguish whether progressive clinical deterioration in the early ART group was secondary to CM complications or to CM-IRIS, which might have resulted in a degree of ascertainment bias, with under-detection of early IRIS events. In contrast, the CM-IRIS case definition performed well for later-occurring IRIS events, in cases where the patients experienced clinical improvement, started to receive antiretroviral treatment, and subsequently showed clinical deterioration. In addition, participants who succumbed in both the early and the deferred ART groups were more likely to have developed IRIS, and therefore our calculations of CM-IRIS incidence may not be precisely accurate.

The risk factors associated with CM-IRIS development include an advanced state of immunosuppression (CD4+ T-cell count less than 50 cells/μL, CD4/CD8 ratio <0.15), high cryptococcal fungal burden at ART initiation, rapid decrease in viral load after ART initiation, and early ART initiation (26–28). Low CSF cellular counts, low inflammatory marker levels, and low IFN-γ response at baseline were also associated with an increased risk of paradoxical IRIS (29). A study by Jarvis et al., observed that IRIS development was associated with excessive polarization toward a T-helper type 1 (Th1) response against cryptococcal antigens after ART initiation, via baseline CSF cytokine analysis (19). Paucity of CSF immune responses during CM has been identified as a major risk factor for CM-IRIS. An inability to mount an effective interferon-driven antiviral immune response, accompanied by a systemic granulocyte pro-inflammatory signature prior to ART initiation, predisposes patients to the development of CM-IRIS (29). Early ART initiation in CM increases CSF cellular infiltrate, macrophage/microglial activation, and T-helper 2 responses within the central nervous system. This suggests that the increased mortality from early ART in the COAT trial was likely to be immunologically mediated (30).

Our study has several limitations. First, unmeasured study-specific effects due to timing or geographic differences between multicenter study sites may have caused residual confounding. Fortunately, these residual confounding effects were minimized by collection of extensive baseline data. Second, the absence of data on outcomes and key predictors, such as cryptococcal antigen titer, early fungicidal activity, biomarkers at baseline, and CSF opening pressures restricted our ability to accurately use these indices to extract important information relevant to our study. Third, our data were extracted from analysis of a fairly small cohort size, with a relatively narrow follow-up duration, which consequently limits the power of the subgroup analyses to prescribe customized guidance for care of individual patients with CM. Consequently, studies based on larger sample sizes and extended observation durations are warranted in the future.

In summary, our study provides evidence that early ART initiation during hospitalization for CM does not improve overall survival in HIV-positive patients with comorbid CM. Moreover, a trend toward increased all-cause mortality was observed with early initiation of ART in subgroup analysis. Furthermore, early ART initiation appears to be associated with an increase in the frequency of grade 4 adverse events, suggesting that it may be safer to defer initiation of ART in HIV-associated CM patients. Our findings emphasize the need for early diagnosis and treatment of HIV infection, preferably before patients present with advanced disease and life-threatening opportunistic infections such as CM.

## Data Availability Statement

The original contributions presented in the study are included in the article/Supplementary Material, further inquiries can be directed to the corresponding author/s.

## Ethics Statement

The studies involving human participants were reviewed and approved by the Ethics Committee of Chongqing Public Health Medical Center (2019-003-02-KY). The patients/participants provided their written informed consent to participate in this study.

## Author Contributions

The study was conceived and designed by Y-KC and ML. ML, JY, J-MN, J-HY, T-TY, G-QZ, JL, and Y-MQ performed the component clinical trials. HC and TZ analyzed data. TZ and X-lX wrote the manuscript, with editorial input from all authors. VH, Y-qL, and TZ edited and revised the article. All authors contributed to the article and approved the submitted version.

## Conflict of Interest

The authors declare that the research was conducted in the absence of any commercial or financial relationships that could be construed as a potential conflict of interest. The handling editor declared a shared affiliation with one of the authors HC at the time of review.

## Publisher's Note

All claims expressed in this article are solely those of the authors and do not necessarily represent those of their affiliated organizations, or those of the publisher, the editors and the reviewers. Any product that may be evaluated in this article, or claim that may be made by its manufacturer, is not guaranteed or endorsed by the publisher.

## Abbreviations

CM: cryptococcal meningitis
ART: antiretroviral therapy
IRIS: immune reconstitution inflammatory syndrome
OIs: opportunistic infections
CSF: cerebrospinal fluid
AST: aspartate aminotransferase
ALT: alanine aminotransferase
ULN: upper limit of normal
CK: serum creatine phosphokinase
TBIL: total bilirubin
Hb: hemoglobin
PLT: platelet count
AMS: blood amylase
WBC: white blood cell count
N: neutrophil count
Scr: serum creatinine
TDF: tenofovir disoproxil fumarate
3TC: lamivudine
EFV: efavirenz
INSTIs: non-nucleoside reverse transcriptase inhibitors
NRTIs: nucleoside reverse transcriptase inhibitors
PIs: protease inhibitors
FIs: fusion inhibitors
CI: Confidence interval
COAT trial: Cryptococcal Optimal ART Timing trial
CNS: central nervous system
IQR: interquartile range
BMI: body mass index
ICP: intracranial pressure
AmB: amphotericin B
5FC: flucytosine
FCZ: fluconazole
LipAmB: liposomal amphotericin B
VCZ: voriconazole.
