# Supplementary material for: The Effect of Early vs. Deferred Antiretroviral Therapy Initiation in HIV-Infected Patients With Cryptococcal Meningitis: A Multicenter Prospective Randomized Controlled Analysis in China
Source: Front Med (Lausanne). 2021 Nov 19;8:779181. doi: 10.3389/fmed.2021.779181 (PMC8639871; doi:10.3389/fmed.2021.779181)
Supplement: Supplementary file 1 [file Data_Sheet_1.DOCX]

**Table S1.** Comparison of baseline characteristics between the early ART-group (<4W) and the deferred ART-group (>6W)

| Characteristic | Deferred ART (>6W) | Early ART (<4W) | *p*-value |
| --- | --- | --- | --- |
| Age, median years (IQR) | 37 (30.5,63.5) | 51 ( 36,60.8) | 0.283* |
| Male, sex, n (%) | 15 (88.2%) | 15 (62.5%) | 0.085# |
| BMI, mean± SD | 20.8 (18.9,22.0) | 20.8 (18.9,22.1) | 0.863* |
| **Symptom** |  |  |  |
| Headache, n (%) | 11 (64.7%) | 16 (66.7%) | 0.896 |
| Fever, n (%) | 10 (58.8%) | 13 (54.2%) | 0.767 |
| Nausea, n (%) | 6 (35.3%) | 9 (37.5%) | 0.885 |
| Vomiting, n (%) | 4 (23.5%) | 8 (33.3%) | 0.729# |
| Impaired consciousness, n (%) | 2 (11.8%) | 5 (20.8%) | 0.679# |
| Stiff neck, n (%) | 4 (23.5%) | 8 (33.3%) | 0.729# |
| **Routine blood tests** |  |  |  |
| WBC, median ×10^9^/L(IQR) | 4.0 (3.0,5.1) | 3.8 (2.5,5.4) | 0.916* |
| Hemoglobin, mean ×g/L± SD | 108.1 ± 24.7 | 96.9 ± 15.9 | 0.113 |
| Platelets, mean×10^9^/L) ± SD | 168.0 ± 67.2 | 178.7 ± 74.2 | 0.639 |
| **Blood biochemistry** |  |  |  |
| TBIL, median µmol/L(IQR) | 7.5 (5.6,10.8) | 8.1 (7.3,10.3) | 0.427* |
| ALT, median U/L(IQR) | 23.0 (14.0,41.7) | 27 (17.7,60.8) | 0.327* |
| AST, median U/L(IQR) | 21.0 (17.5,49.5) | 26.5 (18.0,55.5) | 0.587* |
| Urea, median mmol/L(IQR) | 5.0 (3.3,6.3) | 4.2 (2.8,5.6) | 0.334* |
| Creatinine, median µmol/L(IQR) | 70.1 (56.9,80.2) | 59.7 (47.6,71.0) | 0.057* |
| Other OIs |  |  |  |
| Pulmonary tuberculosis, n (%) | 4 (16.7%) | 1 (11.8%) | 1.0# |
| Pneumocystis pneumonia, n (%) | 3 (17.6%) | 2 (8.3%) | 0.633# |
| *Cytomegalovirus* infection, n (%) | 2 (11.8%) | 1 (4.2%) | 0.560# |
| **CSF profile** |  |  |  |
| ICP, median mm H_2_O (IQR) | 234.7 ± 81.4 | 277.9 ± 84.3 | 0.109 |
| CSF WBC, median×10^6^/L ( IQR) | 44 (9.5,83.5) | 8.0 (4.5,43.0) | 0.068* |
| CSF glucose level, median mmol/L ( IQR) | 2.4 (1.5,2.9) | 2.9 (1.3,3.5) | 0.302* |
| CD4+, median cells/μL (IQR) | 36.0 (6.5,56.0) | 22.0 (13.0,47.5) | 0.916* |
| CD4/CD8 ratio | 0.07 (0.03,0.1） | 0.09 (0.02,0.2) | 0.307* |
| HIV RNA, log10 copies/mL (IQR) | 5.1 ± 0.8 | 5.5 ± 0.6 | 0.064 |
| **Treatment strategy, n (%)** |  |  |  |
| ART regimens containing INSTIs | 11 (64.7%) | 16 (66.7%) | 0.896 |
| AmB/LipAmB as induction antifungal therapy | 15 (88.2%) | 20 (83.3%) | 1.0 |

Data are presented as n (%), mean (± SD) for normally distributed data, or median (IQR) for non-normally distributed data. *p*-values are calculated via χ^2^ tests for categorical variables, and via Student’s t-tests for continuous variables, unless otherwise specified. * Mann-Whitney U test was used. # indicates calculations using the Fisher exact test.

**Table S2.** Grade 3-4 Adverse Events Listing

|  | Intention-to-Treat Population | | | |  | Per-Protocol Population | | | | | |
| --- | --- | --- | --- | --- | --- | --- | --- | --- | --- | --- | --- |
|  | Deferred ART  (n= 55) | Early ART  (n=47) | | *p* | | | Deferred ART  (n= 41) | | Early ART  (n= 37) | | *p* |
| Clinical symptoms, by participant |  | |  | |  |  | |  | |  | |
| Neurological events | 3(5.5%) | | 5(10.6) | | 0.465# | 3(7.3%) | | 5(13.5%) | | 0.466# | |
| Respiratory failure | 4(7.3%) | | 5(10.6%) | | 0.729# | 2(4.9%) | | 5(13.5%) | | 0.247# | |
| Rash-morbilliform | 1(1.8%) | | 0(0%) | | 1.0# | 1(2.4%) | | 0(0%) | | 1.0# | |
| Pyrexia | 2(3.6%) | | 0(0%) | | 0.498# | 2(4.9%) | | 0(0%) | | 0.495# | |
| Routine laboratory tests | | |  | |  |  | |  | |  | |
| Leukopenia | 3(5.5%) | | 2(4.3%) | | 1.0# | 2(4.9%) | | 2 (5.4%) | | 1.0# | |
| Anemia | 6(10.9%) | | 12(25.5%) | | 0.053 | 5(12.2%) | | 10(27%) | | 0.097 | |
| Thrombocytopenia | 1(1.8%) | | 4(8.5%) | | 0.178# | 1(2.4%) | | 4(10.8%) | | 0.184# | |
| Hypokalemia | 6(10.9%) | | 8(17%) | | 0.371 | 6(14.6%) | | 8(21.6%) | | 0.422 | |
| Liver abnormality | 3(5.5%) | | 3(6.5%) | | 1.0# | 3(7.3%) | | 3(8.1%) | | 1.0# | |
| Renal abnormality | 6(10.9%) | | 8(17%) | | 0.371 | 5(12.2%) | | 8(21.6%) | | 0.265 | |

Data are n (%), or median (IQR). # indicates factors were calculated using Fisher’s exact test; otherwise, calculations utilized Pearson’s χ^2^ test.
